# Tuning the Interfacial Reaction Environment for CO_2_ Electroreduction to CO in Mildly Acidic Media

**DOI:** 10.1021/jacs.3c11706

**Published:** 2024-02-13

**Authors:** Xuan Liu, Marc T. M. Koper

**Affiliations:** Leiden Institute of Chemistry, Leiden University, 2300 RA Leiden, The Netherlands

## Abstract

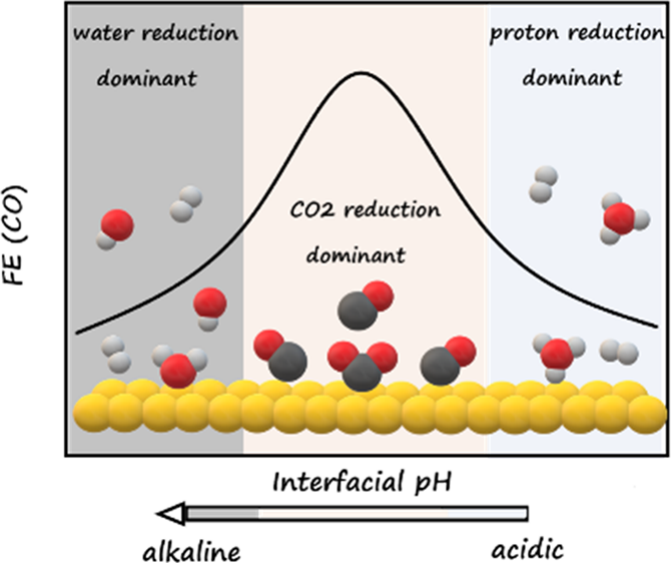

A considerable carbon loss of CO_2_ electroreduction in neutral and alkaline media severely limits its industrial viability as a result of the homogeneous reaction of CO_2_ and OH^–^ under interfacial alkalinity. Here, to mitigate homogeneous reactions, we conducted CO_2_ electroreduction in mildly acidic media. By modulating the interfacial reaction environment via multiple electrolyte effects, the parasitic hydrogen evolution reaction is suppressed, leading to a faradaic efficiency of over 80% for CO on the planar Au electrode. Using the rotating ring-disk electrode technique, the Au ring constitutes an in situ CO collector and pH sensor, enabling the recording of the Faradaic efficiency and monitoring of interfacial reaction environment while CO_2_ reduction takes place on the Au disk. The dominant branch of hydrogen evolution reaction switches from the proton reduction to the water reduction as the interfacial environment changes from acidic to alkaline. By comparison, CO_2_ reduction starts within the proton reduction region as the interfacial environment approaches near-neutral conditions. Thereafter, proton reduction decays, while CO_2_ reduction takes place, as the protons are increasingly consumed by the OH^–^ electrogenerated from CO_2_ reduction. CO_2_ reduction reaches its maximum Faradaic efficiency just before water reduction initiates. Slowing the mass transport lowers the proton reduction current, while CO_2_ reduction is hardly influenced. In contrast, appropriate protic anion, e.g., HSO_4_^–^ in our case, and weakly hydrated cations, e.g., K^+^, accelerate CO_2_ reduction, with the former providing extra proton flux but higher local pH, and the latter stabilizing the *CO_2_^–^ intermediate.

## Introduction

The electroreduction of CO_2_ driven by sustainable energy is envisaged to be an important stride toward a carbon-neutral cycle.^1−4^ In such a cycle, waste or air-captured CO_2_ is reduced back to a broad spectrum of valuable feedstocks, among which carbon monoxide (CO) is of primary interest, due to its economic viability and spectrum of chemical applications.^5^ As an important intermediate and building block, CO can be readily reduced to multicarbon products, either by electroreduction^6^ or by the thermocatalytic Fischer–Tropsch process.^7^

The electrocatalytic CO_2_ reduction reaction (CO2RR) to CO is commonly operated in neutral or alkaline bicarbonate electrolytes to suppress the parasitic hydrogen evolution reaction (HER). Extensive efforts have been devoted to designing better CO2RR catalysts.^3,4^ Still, considerable carbon loss during the CO2RR due to its conversion to carbonate in an alkaline reaction environment severely limits its practical feasibility. According to various studies so far, it is commonly believed that HCO_3_^–^ and CO_3_^2–^ are not directly reduced on a bare gold electrode,^8,9^ but rather function as a potential carbon supply^8^ for CO2RR and as a proton donor^10,11^ for HER. Hence, the generation of HCO_3_^–^ and CO_3_^2–^ by homogeneous reactions severely compromises the carbon efficiency of CO2RR. This “carbonate problem” has been considered by Rabinowitz and Kanan et al.^12^ to be the biggest obstacle to real-world applications of CO2RR. The “carbonate problem” means that, in addition to being reduced on the electrode surface, CO_2_ is also consumed by the electrogenerated OH^–^ to produce HCO_3_^–^ or CO_3_^2–^ according to reaction eqs 1 and 2, which are thermodynamically favorable in alkaline media:
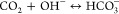
1
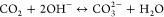
2

Due to the proton-coupled electron transfer nature of CO2RR and HER, every electron transferred at the interface generates one OH^–^, resulting in a local alkaline environment and the conversion of CO_2_ to HCO_3_^–^ or CO_3_^2–^ in the electrolyte, decreasing the carbon efficiency and multiplying the cost for downstream processing, e.g., regenerating CO_2_ from carbonate. In that sense, the larger the current density, the lower the carbon efficiency, thereby largely limiting the industrial practicality of CO2RR.

To circumvent the “carbonate problem”, CO2RR in acidic media has recently emerged as a possibly attractive alternative.^13,14^ However, the low bulk pH brings about the strong competition from HER due to proton reduction (as opposed to HER from water reduction in neutral and alkaline media), lowering the FE of CO2RR. Studies on acidic CO2RR have focused on promoting CO2RR and suppressing HER in acidic media, mainly by suitable design of the electrolyzer configuration,^15^ catalyst engineering,^16−21^ and electrolyte modification.^13,22−25^ Oßkopp et al.^15^ studied the FE and the local pH of a tin-oxide catalyst on gas-diffusion electrodes (GDEs) in different cell configurations, and found that a divided cell with a zero-gap anode is capable of producing undissociated formic acid, suggesting that the (local) pH remains lower than the p*K*_a_ of formic acid (p*K*_a_ = 3.77). Also, tuning the structure and composition of catalysts may effectively enhance the activity and selectivity of CO2RR. Confinement in nanostructures has been suggested to increase the local concentration of alkali cations and inhibit proton diffusion or kinetically reduce the local proton concentration, leading to a higher FE of CO2RR,^16,17^ while bimetallic catalysts have been suggested to weaken H binding which when combined with a high affinity for CO2RR reaction intermediates promotes C–C coupling.^19^ Organic polymer-modified electrodes change the hydrophobicity of the electrode and the proton activity near the surface, hence tailoring the proton transfer rates at the interface.^21,26^ Besides, electrolyte conditions have a profound effect on the interfacial reaction environment.^6,27−29^ Weakly hydrated cations such as K^+^ and Cs^+^ are indispensable for CO2RR in acidic media^14,17,30,31^ due to their stabilization of reaction intermediates through electrostatic interaction.^25,30,32^ Alkali cations have also been proposed to modify the local electric field in the double layer,^23,32^ buffer the interfacial pH under an alkaline local environment,^33−35^ and suppress the migration of hydronium ions to the surface.^23,24^ Indeed, mass transport crucially influences the competition between CO2RR and HER. Bondue et al.^13^ used differential electrochemical mass spectroscopy (DEMS) with a dual-compartment flow cell to study CO2RR on Au electrodes in mildly acidic media and proposed that HER can be suppressed if the rate of CO2RR matches the mass-transport rate of protons, as OH^–^ formation from CO2RR is then sufficient to neutralize protons near the electrode before they discharge to produce hydrogen. The applicability of this idea in practical GDE geometries has been verified by Monteiro et al.^22^; the FE of CO2RR reaches over 80% at current densities up to 200 mA cm^–2^ on 10 cm^2^ Au GDEs in acidic media.

Given the great importance of the near-electrode electrolyte conditions and especially interfacial pH, during acidic CO2RR, systematic studies with operando techniques are important to uncover real-time information about the interfacial reaction environment and devise suitable strategies to tune the local environment. The rotating ring-disk electrode (RRDE) technique is a powerful electroanalytical tool in studies of CO2RR. The well defined mass-transport conditions of RRDE renders the ring electrode a quantitative collector of the two main products generated from the disk electrode during CO2RR, namely CO and OH^–^, as has been shown in our previous works.^34,36−38^ As a selective CO-producing^9^ and an excellent CO-oxidation^39^ catalyst, a Au ring and disk were used throughout the study, so that only CO and H_2_ were generated on the disk and CO was oxidized exclusively on the Au ring,^36^ enabling us to deconvolute CO2RR and HER in the RRDE setup. Furthermore, we believe that the pH (and CO) sensor being remote from the reaction interface is one of the advantages of our device, as the measurements are conducted without disturbing the reaction environment. Due to the good time resolution of the RRDE pH sensor, we are able to trace the evolution of the interfacial environment during transient techniques, e.g. cyclic voltammetry,^34,37^ in combination with well defined mass-transport conditions. It is very difficult to detect the interfacial pH under the same reaction conditions as RRDE by other techniques, such as in situ spectroscopies. pH measurements by in situ spectroscopy are mostly performed with special electrochemical cells, which greatly impacts the mass transport. Moreover, in spectroscopy, the ratio of the integrated peak areas between CO_2_ and HCO_3_^–^ is commonly used to determine the interfacial pH during CO2RR.^35,40^ This compromises the time resolution of the measurements, as the equilibrium between CO_2_ and HCO_3_^–^ is established slowly,^41^ making it more difficult to record the interfacial pH accurately during cyclic voltammetry. A disadvantage of the RRDE technique is that it measures the pH at the ring, which then needs to be converted mathematically to the pH at the disk. This requires a model, i.e., knowledge of all relevant acid–base equilibria in the system, which in a complex environment may lead to inaccuracies if certain equilibria are not included. An additional disadvantage of the RRDE method may be that it is less local or interface-specific than the vibrational methods. For instance, sum frequency generation spectroscopy can estimate the local pH on a molecular length scale.^42^ RRDE averages out the pH gradients in the lateral directions. In the direction perpendicular to the electrode, RRDE measures the pH on a local scale that is consistent with a continuum description, which is certainly less than molecular but still relevant to describe local concentration gradients.

In this work, we probe the interfacial pH using a modified Au ring electrode and measure both the FE of the CO and the interfacial pH during acidic CO2RR on a Au disk electrode. The measurement of reaction selectivity and interfacial pH allows us to study in quantitative detail the relation between the interfacial environment and the relevant reactions: specifically, the interaction between the CO2RR and two main branches of the HER, namely, proton reduction and water reduction, can be elucidated. Additionally, the influence of different electrolyte conditions, such as anion identity, cation identity, and mass transport rate, on the interfacial environment and the reaction selectivity can be explored quantitatively. A proper protic anion, a weakly hydrated cation, and slow mass-transport conditions are demonstrated to improve the FE of the CO2RR on a Au electrode in acidic media. Since these parameters primarily reflect the electrolyte, we expect these conclusions to generalize to other electrode materials and structures.

## Experimental Section

### Chemicals and Materials

Electrolytes were prepared with ultrapure water (>18.2 MΩ cm, Millipore Milli-Q) and the following chemicals: Li_2_SO_4_ (>99.99%, trace metal basis, Sigma-Aldrich), Na_2_SO_4_ (anhydrous, 99.99% Suprapur, Sigma-Aldrich), K_2_SO_4_ (>99.99%, trace metal basis, Sigma-Aldrich), NaClO_4_·H_2_O (>99.99%, trace metal basis, Sigma-Aldrich), NaH_2_PO_4_(99.998%, trace metals basis, Sigma-Aldrich), and H_2_SO_4_ (96% Suprapur, Merck). The pH of the electrolytes was adjusted with H_2_SO_4_ (96% Suprapur, Merck) and HClO_4_ (70% Suprapur, Merck,). All electrolytes were purged with either Ar (6.0 purity, Linde, 20 min) or CO_2_ (4.5 purity, Linde, 20 min) before experiments. All the electrochemical experiments were performed in homemade single-compartment electrochemical cells, controlled by a four-channel Biologic potentiostat (VSP-300) and a modulated speed rotator (Pine Research). A three-electrode system was employed in all the electrochemical measurements with a ring-disk electrode (E6R1 ChangeDisk, PEEK Shroud, Pine Research), a Au wire (0.5 mm diameter, MaTeck, 99.9%), and a Ag/AgCl electrode (RE-1B, 3 M NaCl, Biologic, inserted in a Luggin capillary) as the working electrode, counter electrode, and reference electrode, respectively. The electrochemical cells and other glassware were kept in a KMnO_4_ solution (1 g L^–1^ KMnO_4_ in 0.5 M H_2_SO_4_) overnight. Before experiments, they were immersed in dilute piranha to remove the generated MnO_*x*_ and the residual KMnO_4_, followed by rinsing and boiling in ultrapure water five times.

### Preparation and Modification of the Electrodes

The RRDE tip was polished with 3 μm, 1 μm, and 0.25 μm diamond suspension (MetaDi, Buehler), respectively, with the Au disk (*D* = 5 mm) inserted in the Au ring matrix (*D*_inner_ = 6.5 mm, *D*_outer_ = 7.5 mm). It was sonicated in ethanol and ultrapure water for 5 min between each polishing step. Then, the Au ring and disk electrodes were short-circuited and electropolished in 0.1 M H_2_SO_4_ by cycling between 0 and 1.75 V vs RHE at 1 V s^–1^ (Ar-saturated) for 200 times, followed by cyclic voltammetry under the same condition at 100 m V s^–1^ on Au ring and disk electrode separately, to characterize the surface and calculate the electrochemical surface area (ECSA) by dividing the charge of the Au oxide reduction peak by the charge density of a Au oxide monolayer (386 μC cm^–2^) (see Figure S1 in the Supporting Information).

### Interfacial pH Measurements

The pH sensor coupled with RRDE has been developed in our group; the details are given in our previous works.^34,37^ Briefly, the Au ring electrode was modified by a monolayer of 4-nitrothiophenol (4-NTP) by dipping the RRDE tip (with a Au ring and a Teflon disk) in a 1 mM ethanal-dissolved 4-NTP (80%, Merck) solution for 20 min. The 4-NTP is then converted to the pH-sensing couple 4-hydroxylaminothiophenol/4-nitrosothiophenol (4-HATP/4-NSTP), whose redox potential is pH-dependent by cyclic voltammetry in 0.1 M H_2_SO_4_ from 0.68 to 0.11 V vs RHE at 100 mV s^–1^.

During the pH measurements, the potential of the Au disk was swept negatively from 0 V vs RHE in different electrolytes at 2 mV s^–1^. Simultaneously, the peak potentials of the 4-HATP/4-NSTP redox couple on the ring were continuously monitored by cyclic voltammetry at 200 mV s^–1^. The peak potentials shift as the interfacial environment of the ring electrode evolves, with the reactions occurring on the disk electrode. Hence, the cycling range of the pH sensor was tuned if necessary. For instance, during the measurements in 0.1 M Na_2_SO_4_ (pH = 3), the potential window on the ring was kept as −0.05 to 0.35 V vs Ag/AgCl from the start to −0.5 V vs RHE on the disk. It was then changed to −0.15 to 0.25 V vs Ag/AgCl as the interfacial environment became less acidic. The gases (CO_2_ or Ar) were kept purging into the electrolyte during the measurements to eliminate any interference from oxygen. Details of the calculations of the interfacial pH at the disk are explained in the Supporting Information.

All pH data reported in the following are calculated pH data for the disk electrode. The actually measured ring pH data are collected in the Supporting Information, section “Ring pH Data”.

### Faradaic Efficiency Measurements

The CO-sensing method, based on RRDE, was developed by our group and has been applied in multiple investigations. The detailed procedure is described in the previous publications.^11,36,38^ In this work, the Faradaic efficiency measurement was carried out subsequently with the interfacial pH measurement on the same Au disk electrode. After the pH measurement, the Au disk was disassembled from the RRDE tip, and the Au ring matrix was coupled with a Teflon disk to be repolished and sonicated following the procedure mentioned above to remove the pH-sensing monolayer. Next, the Au disk was reassembled in the Au ring matrix, followed by electropolishing and characterization as aforementioned. To eliminate the interference from bubbles during measurements, the PEEK shroud and the Teflon spacer between the ring and disk electrode were coated with dopamine to increase their hydrophilicity by immersing the RRDE tip in 0.1 M NaHCO_3_ dissolved 2 g/L dopamine hydrochloride for 1 h at about 55 °C, with the rotation rate at 450 rpm. Then, the Au ring and disk electrode were electropolished in 0.1 M H_2_SO_4_ again to remove the dopamine residue from the Au electrode surface. Subsequently, the Au ring and disk electrodes were characterized again in 0.1 M H_2_SO_4_. The cyclic voltammograms derived agree well with the ones obtained before the dopamine coating and the ones before interfacial pH measurements, suggesting the complete elimination of the dopamine residue and no detectable variation of the electrode surface during the process (Figure S1). During the FE measurement, the Au disk was cycled from 0 to – 1.5 V versus RHE in different electrolytes at 2 mV s^–1^, while the Au ring potential was set as 1 V versus RHE to oxidize the CO generated on the disk electrode. At this potential, CO oxidation is diffusion-limited in bicarbonate solution. In a phosphate-containing solution, the current is slightly below the diffusion-limited current at 1 V, approaching diffusion limitation at higher (interfacial) pH.^43^ This means that in solution with more strongly adsorbing anions (such as sulfate and phosphate), the actual CO concentration may be slightly underestimated, though we expect the error to be small if the interfacial pH is high. The apparent collection efficiency of the ring was determined at the end of the measurements to inspect if there was any deviation from the theoretical value due to changes in geometry during assembly of the tip. The apparent collection efficiency was measured in 5 mM K_3_Fe(CN)_6_ dissolved in 0.1 M NaHCO_3_, during which the disk was cycled from 0.27 to 1.27 V vs RHE, while the ring potential was set to 0.96 V vs RHE. The collection efficiency was determined for each rotation rate and was calculated according to eq 3.
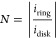
3Details of the calculations of the Faradaic efficiency are explained in the Supporting Information.

## Results and Discussion

For each interfacial pH measurement, the pH is recorded by the highly sensitive pH sensor on the ring and then converted to the pH disk according to the equations originally derived by Albery and Calvo.^44^ We also introduced a buffering correction in the calculation to compensate for the deviation caused by the presence of buffering species. Detailed calculations and the pH profiles of RRDE in different electrolytes are explained in the Supporting Information. We note that in this work we semiquantitatively correlate the changes in the interfacial pH values during cyclic voltammetry with different electrolyte parameters, such as anion identity, cation identity, and rate of mass transport, instead of asserting absolute accuracy of individual pH values.

**Figure 1:**
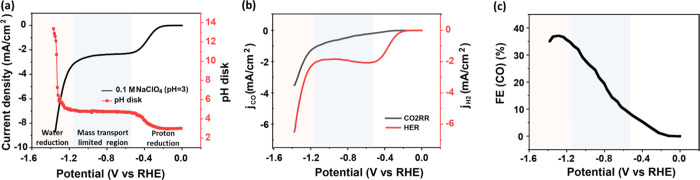
(a) Variation of the interfacial pH recorded during cyclic voltammetry in 0.1 M CO_2_-saturated NaClO_4_ with a bulk pH of 3, at 2 mV s^–1^ and a rotation rate of 2500 rpm: the black line and the red curve refer to the current density and to the corresponding interfacial pH during the negative-going scan, respectively. (b) The partial current density of CO2RR (black curve) and HER (red curve) during the cyclic voltammetry from (a). (c) The Faradaic efficiency of CO during the cyclic voltammetry as derived from (a). The potentials in all figures have been converted to the RHE scale using the bulk pH.

Interfacial pH and FE measurements were first carried out in 0.1 M NaClO_4_ with the bulk pH adjusted to 3. As depicted in Figure 1a, an increase in current density is observed at – 0.3 V, which is ascribed to proton reduction. Depending on the proton source, HER in an aqueous solution takes place through either the proton reduction reaction (eq 4) or the water reduction reaction (eq 5). 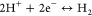
4
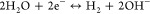
5As only traces of CO are detected there (Figure 1b), the first region is dominated by the proton reduction reaction. Between −0.6 and −1.1 V, the current corresponds to mass transport-limited proton reduction. However, as illustrated in Figure 1b, the current due to CO2RR discernibly increases, with a corresponding decrease in HER current. Interestingly, the total current remains constant. During the mass transport-limited proton reduction, the interfacial pH near the disk electrode is ca. 5. This pH is lower than the pH of 7 measured previously in the absence of CO_2_ (though in sulfate electrolyte), which must be due to a buffering effect of the CO_2_. Since CO2RR is a cation-coupled electron transfer reaction,^30^ Bondue et al.^13^ have previously argued that OH^–^ is generated from CO2RR (eq 6), which reacts with “incoming” protons. 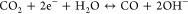
6This explains the correspondence between the CO2RR increase and HER decrease. With increasingly negative potential, the CO2RR rate increases, and more protons are neutralized by OH^–^ before reaching the surface, thereby suppressing the proton reduction further. At around −1.2 V, water reduction initiates, causing a sharp increase in the total current density and a decay of the FE for CO (Figure 1c), even though the partial current density of the CO2RR still rises.

With increasing current density in the water reduction region, the interfacial pH rises rapidly from −1.3 V, due to the small buffer capacity of the electrolyte, as there is only 35 mM of carbonaceous buffering species (CO_2_: buffer range 5.3–7.3; HCO_3_^–^: buffer range: 9.3–11.3) in the bulk phase. This is in agreement with previous studies^19,45−47^ showing that the interfacial environment during CO2RR and HER in weakly buffered acid turns highly alkaline. The interfacial pH is most effectively lowered by increasing the concentration of buffering species. Our previous work^34^ showed that the interfacial pH during CO2RR decreases from 11 to 9 as the concentration of HCO_3_^–^ increases from 0.1 to 0.5 M. The decline in the FE for CO is in agreement with the studies in neutral bicarbonate media: the FE of CO on a planar Au electrode reaches its maximum just before the onset potential of water reduction, with the interfacial environment turning alkaline.^11,36^ While CO2RR can suppress proton reduction under appropriate conditions, it does not compete effectively with water reduction at these negative potentials. This is partially due to the substantial consumption of CO_2_ by chemical reactions via eqs 1 and 2 under the highly alkaline interfacial environment during water reduction.

**Figure 2:**
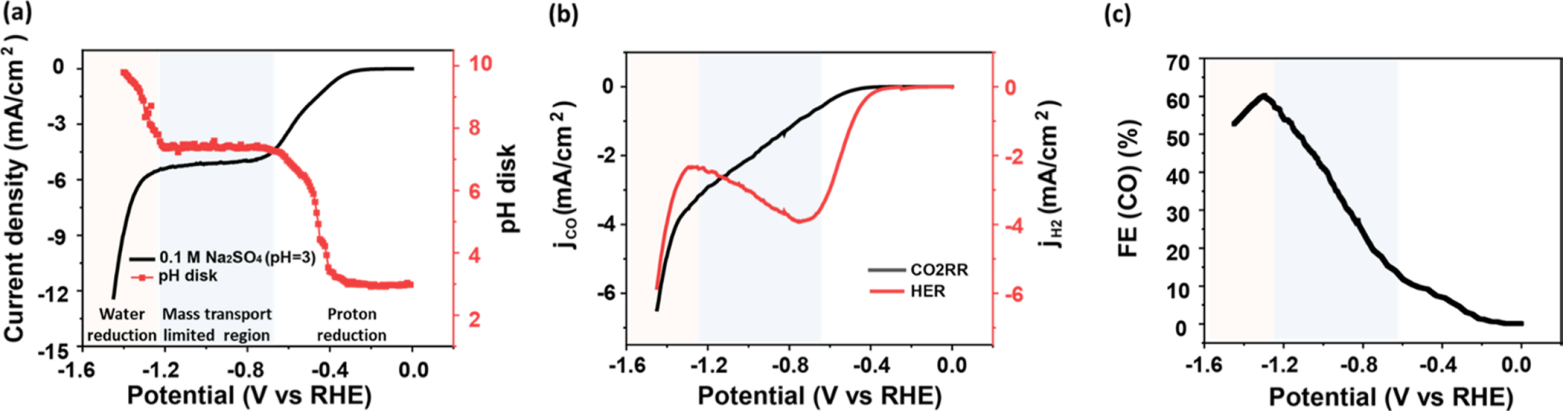
(a) Variation of the interfacial pH recorded during cyclic voltammetry at 2 mV s^–1^ and a rotation rate of 2500 rpm in 0.1 M CO_2_-saturated Na_2_SO_4_ with a bulk pH of 3: the black line and the red curve refer to the current density and to the corresponding interfacial pH during the negative-going scan, respectively. (b) The partial current density of the CO2RR (black curve) and HER (red curve) during the cyclic voltammetry from (a). (c) The Faradaic efficiency of CO during the cyclic voltammetry as derived from (a).

Interfacial pH and FE measurements were also performed in 0.1 M Na_2_SO_4_ (acidified to pH 3) to study the effect of a different anion. As in NaClO_4_, there are discernible regions for proton and water reduction, as shown in Figure 2a. Compared to results in NaClO_4_, the onset potential of proton reduction in Na_2_SO_4_ has shifted slightly negatively, likely related to the specific adsorption of SO_4_^2–^ on the Au surface.^48^ Surprisingly, a higher limiting current density is obtained in Na_2_SO_4_, with an identical bulk pH as NaClO_4_. Due to this larger current density, by the end of the proton reduction region, the interfacial pH has increased up to 7, indicating a closely neutral interfacial environment, which could be beneficial for the CO2RR. This is illustrated in Figure 2b: the current density of the CO2RR in Na_2_SO_4_ is nearly four times larger than that in NaClO_4_, leading to a faster consumption of protons and a remarkable decay in the proton reduction. By the end of the mass transport-limited region, the FE of CO reaches 60% in Na_2_SO_4_, which is about 1.5 x larger than that in NaClO_4_, but it drops quickly as the water reduction starts (Figure 2c).

**Figure 3:**
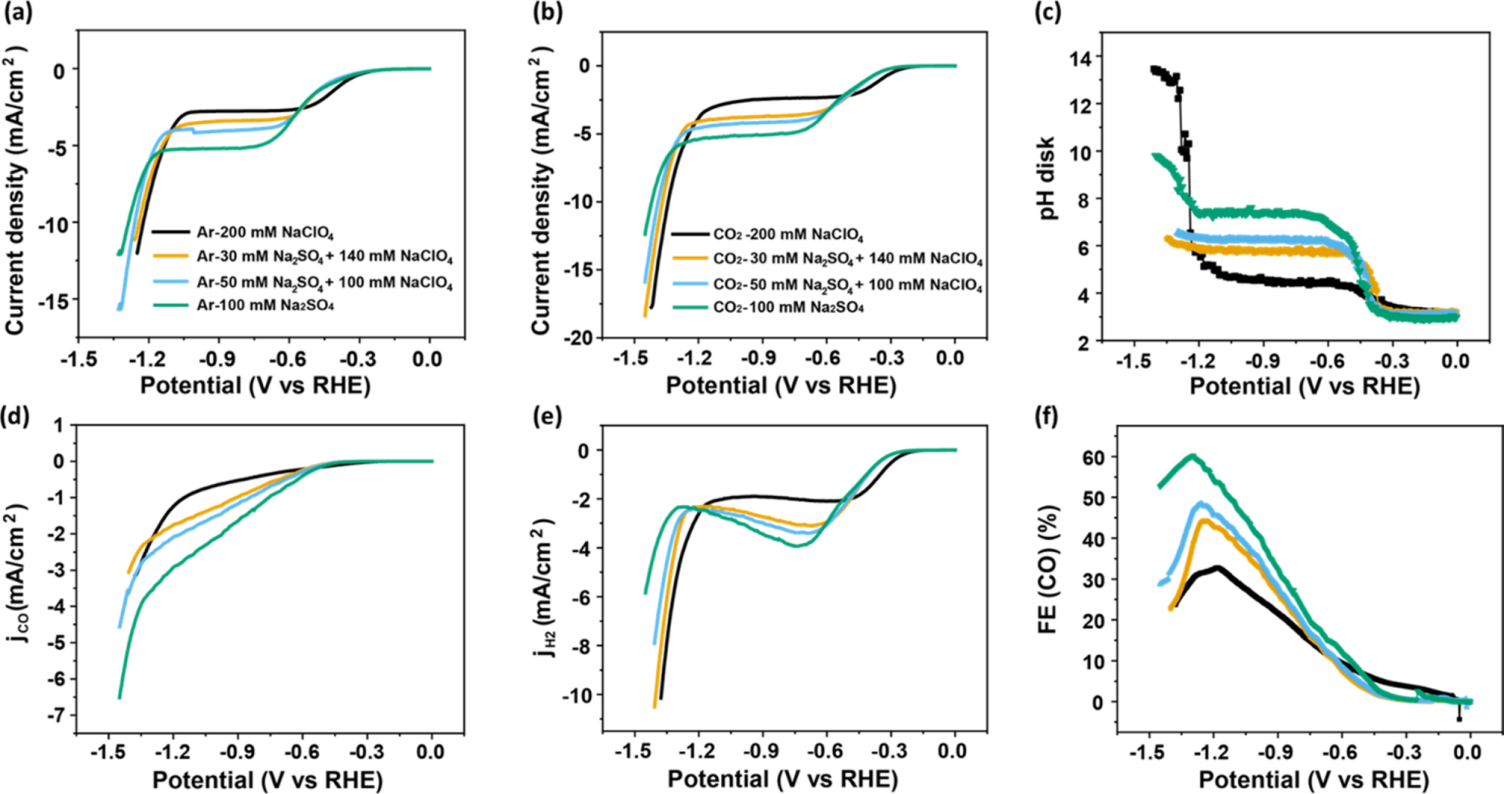
Cyclic voltammograms in sulfate-containing electrolytes with 200 mM Na^+^ and different SO_4_^2–^ concentrations with a bulk pH of 3 at 2 mV s^–1^ and a rotation rate of 2500 rpm under (a) Ar and (b) CO_2_ atmosphere. Variation of (c) the interfacial pH, (d) the partial current density of CO2RR, (e) the partial current density of HER, and (f) the Faradaic efficiency of CO as a function of potential during the cyclic voltammetry from (b).

To carefully inspect this increasing limiting current density in SO_4_^2–^, measurements were performed in electrolytes containing different SO_4_^2–^ concentrations from 0 to 200 mM with a bulk pH of 3. The cation concentrations were kept at 0.2 M by adding different amounts of NaClO_4_. Figure 3a,b depicts the variation of the current densities with SO_4_^2–^ concentration in Ar and CO_2_ atmosphere, respectively. As the limiting current density is determined by the total concentration of the proton sources in the bulk phase, the increase in limiting current density with SO_4_^2–^ concentration in Figure 3a signifies a higher concentration of proton donors in solution, which are not only the hydronium cations but also any conjugated acid in the electrolyte that is able to deprotonate and release protons. Coupling to acid–base equilibria in solution has been shown to give higher mass-transport-limited currents; for a mathematical treatment, see the original work of Koutecky and Levich^49^ and of Rebouillat et al.^50^ In 0.1 M CO_2_-saturated Na_2_SO_4_ at a bulk pH of 3, other than 1 mM hydronium cations, there are 9 mM HSO_4_^–^ in the bulk electrolyte. The effective buffer range of HSO_4_^–^/SO_4_^2–^ is 0.99–2.99. The interfacial pH increases out of this pH range after −0.3 V, making the HSO_4_^–^ here behave more like a proton donor rather than an effective buffer. As the potential shifts negatively, the concentration of protons and HSO_4_^–^ near the interface consistently decrease. As a result of this pH gradient, HSO_4_^–^ dissociates and releases a proton via eq 7. 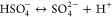
7When reaching the vicinity of the surface, the proton flux from HSO_4_^–^ adds to the overall proton reduction or neutralization of OH^–^ generated from CO2RR. With the added proton flux from HSO_4_^–^, larger proton reduction currents are observed accordingly. Interestingly, this higher mass transport-limited effective proton reduction current also leads to higher interfacial pH, as deduced from the pH measurements on the ring (see Figure S3) and disk (see Figure 3c).

Once the CO2RR starts, the proton reduction decays due to, as mentioned above, CO2RR generating hydroxide ions, which consume the protons that would otherwise contribute to proton reduction. The results indicate that CO2RR increases with the SO_4_^–^ concentration. As shown in Figure 3, with the SO_4_^–^ concentration raised from 30 to 200 mM, the current density of the CO2RR is enhanced by a factor of 2, leading to the FE of the CO increasing from 40 to 60%. The reason for this enhancing effect of the higher SO_4_^–^ concentration on the CO2RR is not entirely clear. It likely has to do with the higher interfacial pH, which may also lead to a higher local concentration of cations., which then promotes CO2RR.

**Figure 4:**
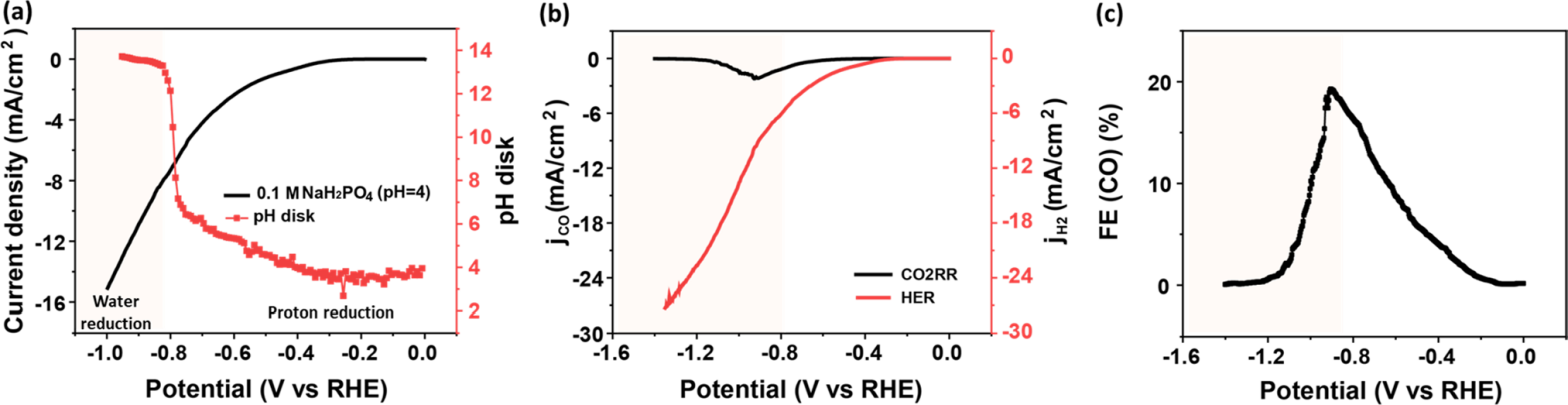
(a) Variation of the interfacial pH recorded during cyclic voltammetry at 2 mV s^–1^ and a rotation rate of 2500 rpm in 0.1 M CO_2_-saturated NaH_2_PO_4_ with a bulk pH of 4: the black line and the red curve refer to the current density and to the corresponding interfacial pH during the negative-going scan, respectively. (b) The partial current density of CO2RR (black curve) and HER (red curve) during the cyclic voltammetry from (a). (c) The Faradaic efficiency of CO during the cyclic voltammetry as derived from (a).

The influence of another typical protic anion, namely, H_2_PO_4_^–^, was also studied, by conducting the same experiments in 0.1 M NaH_2_PO_4_ with a bulk pH of 4. Unlike the results in Na_2_SO_4_ and NaClO_4_, no obvious mass transport-limited region is detected (Figure 4a). This is attributed to the large proton flux brought by 0.1 M H_2_PO_4_^–^, which can contribute more than 0.2 M protons during reactions. As Figure 4b illustrates, with strong support from H_2_PO_4_^–^ (p*K*_a_ = 7.20), the total current density is remarkably larger than that in Na_2_SO_4_ and NaClO_4_. This large current density is mainly due to the high promotion of HER by phosphate anions. The partial current density of HER in NaH_2_PO_4_ increases by two times compared to that in Na_2_SO_4_ and NaClO_4_. Jackson et al.^51^ also reported that the contribution to HER by direct phosphate reduction can outcompete water as the dominant proton source and enable HER activity at neutral pH comparable to that at pH 1. Consequently, the interfacial pH rises continuously with a higher current density. There is 100 mM phosphate buffer species (H_2_PO_4_^–^: buffer range 6.2–8.2; HPO_4_^2–^: buffer range: 11.3–13.3) in the bulk phase. Due to the buffering of H_2_PO_4_^–^, the interfacial pH increases slowly from 5 to 8 with increasing current density. Once depleting H_2_PO_4_^–^ near the interface, the pH increases dramatically and HPO_4_^2–^ starts to buffer, resulting in a pH plateau at around 13. As the interfacial environment becomes highly alkaline, the CO2RR is severely limited. The current density of the CO2RR declines at −0.9 V with a maximum FE of 20%. Hence, one should be very careful when involving buffer species in the electrolyte, because these protic buffer anions not only influence the interfacial pH but are also highly likely to impact the proton–electron transfer to generate hydrogen at the interface.^52^

**Figure 5:**
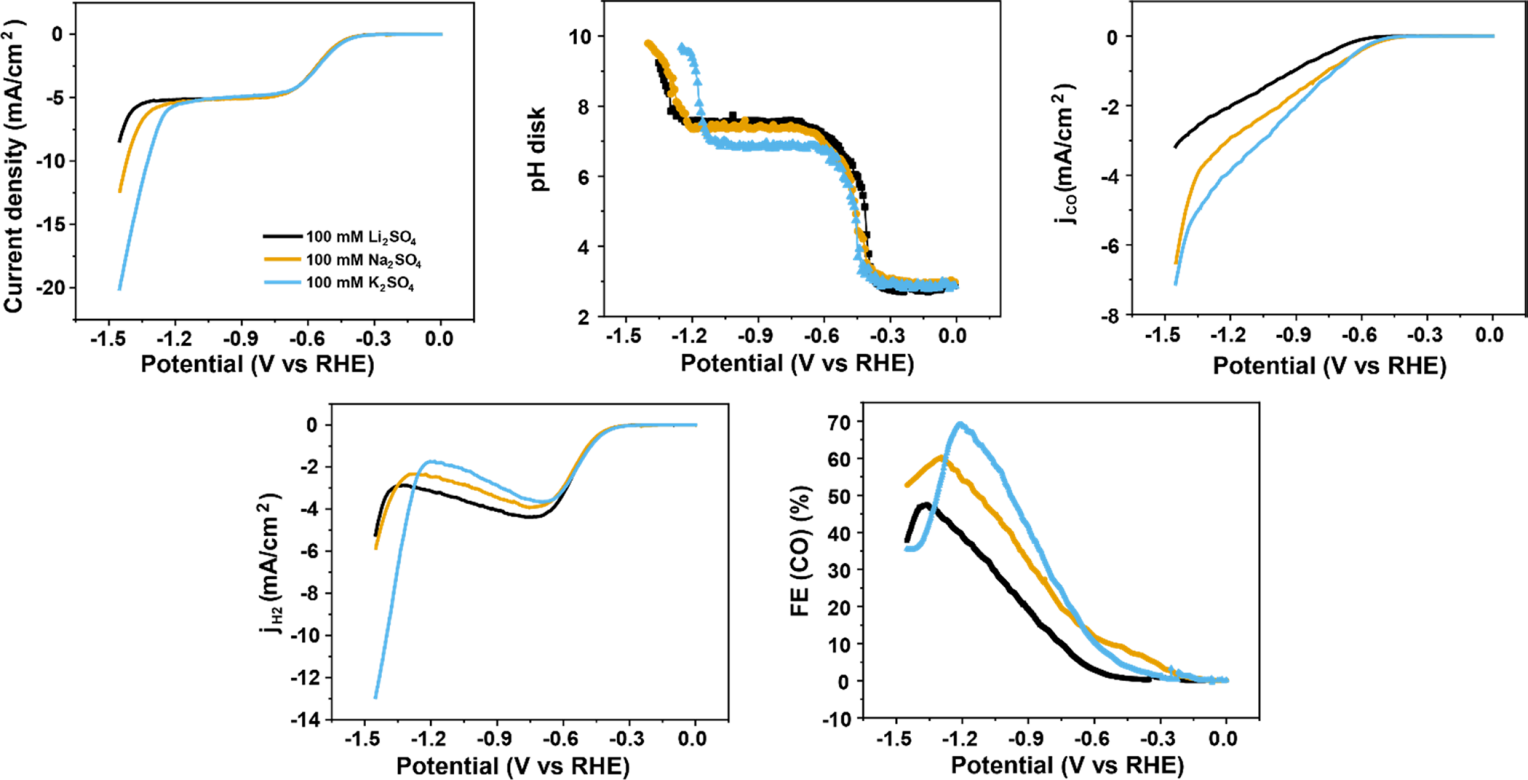
(a) Cyclic voltammograms in CO_2_-saturated 100 mM sulfate with different cation identity with a bulk pH of 3 at 2 mV s^–1^ and a rotation rate of 2500 rpm. Variation of (b) the interfacial pH, (c) the partial current density of CO2RR, (d) the partial current density of HER, and (e) the Faradaic efficiency of CO as a function of potential during the cyclic voltammetry from (a).

The effect of cations in acidic media was investigated by measurements in sulfate electrolytes with different cations (pH = 3). As shown in Figure 5a, results with different cations show a nearly identical proton reduction-dominant region, demonstrating that the proton reduction reaction is independent of cation identity, in agreement with the literature.^25^ Additionally, there is no apparent disparity observed in mass transport-limited regions with different cations, as the cations have no significant influence on the total proton flux in this case. However, as the activity of the CO2RR varies with different cations, the proton reduction current in the mass transport-limiting region is affected indirectly. Figure 5c shows that the CO2RR rate increases from Li^+^ to K^+^, demonstrating that weakly hydrated cations promote CO2RR. Consequently, the activity of CO2RR is highest in K_2_SO_4_, and the proton reduction is suppressed accordingly, leading to the largest FE of 70% in the K^+^-containing electrolyte (Figure 5e). Interestingly, the FE in K^+^ decreases sharply once the water reduction sets in. This is because the higher concentration of weakly hydrated cations near the interface also contributes to a higher activity of water reduction, by stabilizing the transition state of its rate-determining step.^53^ Consequently, the onset potential of water reduction shifts positively from Li^+^ to K^+^, resulting in a corresponding decay of the CO2RR rate. Moreover, the interfacial pH in K^+^ is smaller than that in Na^+^ and Li^+^ in the mass transport-limiting region (Figure 5b), even under the same current density. This can be explained by the theory of cation hydrolysis:^33^ as the hydrated cation locates in proximity to the interface, its hydration shell interacts strongly with the negative charge on the electrode, causing a significant decrease in the p*K*_a_ of the cation (11.64 for Li^+^, 10.26 for Na^+^, 7.95 for K^+^) and facilitating the hydrolysis of the water molecule from the hydration shell to release protons.^33^ Since the interfacial pH here is close to the p*K*_a_ of K^+^, protons are released from the hydration shell of K^+^ and decrease the interfacial pH.

**Figure 6:**
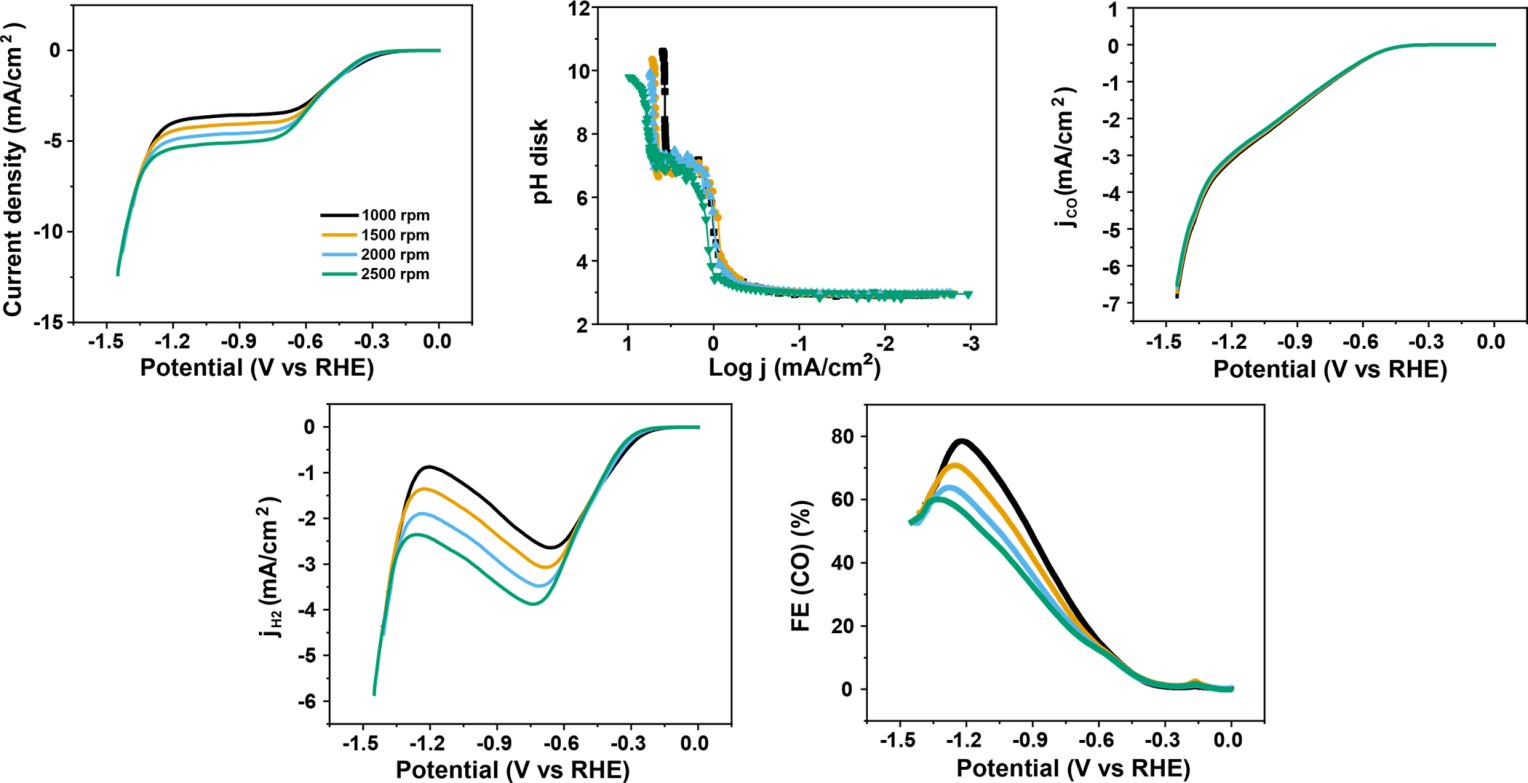
(a) Cyclic voltammograms in CO_2_-saturated 100 mM Na_2_SO_4_ with a bulk pH of 3 at 2 mV s^–1^ with different rotation rates. Variation of (b) the interfacial pH, (c) the partial current density of CO2RR, (d) the partial current density of HER, and (e) the Faradaic efficiency of CO as a function of potential during the cyclic voltammetry from (a).

The effect of mass transport was studied by measurements in 0.1 M Na_2_SO_4_ (pH 3) under different disk rotation rates from 1000 to 2500 rpm. As shown in Figure 6a, mass transport only affects the HER current: there is no effect on the CO2RR current under these conditions.

This observation is compelling evidence for the theory that suggests that protons are not directly involved in CO2RR, but rather that CO2RR is an OH^–^ generating reaction: at higher mass transfer rates, OH^–^ generated from CO2RR can neutralize fewer incoming protons, leading to a lower FE. This effect is opposite to the mass-transport effect in an alkaline environment: FE of CO2RR in neutral or alkaline media increases with mass transport, mainly due to the competing water reduction being suppressed by decreasing interfacial pH.^36^ The results in Figure 6 also illustrate that there is no CO_2_-related mass-transport effect on the CO2RR, as is often claimed in the literature. The observed mass-transport effects are all indirectly related to the mass-transport-sensitive HER.^6^

## Conclusions

In this work, we have shown quantitatively using rotating ring-disk voltammetry how modulation of the interfacial reaction environment during CO2RR in mildly acidic media (pH 3) on planar Au electrodes can generate situations that suppress most of HER, with a correspondingly high Faradaic efficiency (up to 80% under our conditions) and carbon efficiency.

Under acidic conditions, there are three ranges during the reaction process, namely, the proton reduction-dominant region, the proton mass transport-limited region, and the water reduction-dominant region. At mildly negative potential in an acidic interfacial environment, the protons discharge on the surface. The interfacial reaction environment becomes less acidic with an increasing current density. Prior to the depletion of protons, CO_2_RR discernibly increases, signifying the beginning of the second range. Although the total current density is still limited by the proton flux and the interfacial pH keeps constant accordingly, the partial current density of CO2RR increases with increasingly negative potential due to the protons being neutralized by the OH^–^ produced by CO2RR. By the end of the mass transport-limited regime, CO2RR reaches the maximum in FE just before the water reduction initiates. From then on, OH^–^ is formed by water reduction, and the interfacial environment quickly turns more alkaline. CO2RR is inhibited as a result of the considerable depletion of CO_2_ by the reaction with OH^–^.

The interfacial reaction environment can be tuned by anion identity, cation identity, and mass transport. A proper protic anion such as HSO_4_^–^ can supply extra proton flux and tune the interfacial environment to be nearly neutral. Besides, the influence of the cation effect and mass-transport effect in an acidic interfacial environment is different from that in an alkaline interfacial environment since CO2RR competes with different HER branches. In an acidic environment, a weakly hydrated cation such as K^+^ accelerates the CO2RR while barely impacting the competing proton reduction, leading to a higher FE of the CO2RR. However, it decays drastically when reaching an alkaline interfacial environment, as the competing water reduction is also promoted by a weakly hydrated cation. The FE of the CO2RR decreases with enhanced mass transport in an acidic interfacial environment as the rate of OH^–^ generation by the CO2RR cannot keep up with the mass transfer rate of the protons. This mass-transport effect is very different from the situation in an alkaline environment.^36^

Our study sketches the interrelationship between different reactions and the interfacial environment and specifies the interrelationship between CO2RR and two major branches of HER, namely proton reduction and water reduction, respectively. CO2RR is able to outcompete proton reduction under suitable conditions, while water reduction decreases the Faradaic and carbon efficiency of CO2RR.

From our work, we conclude that the CO2RR is far from mass-transport-limited, even in our setup with strong forced convection. We expect that this conclusion may be relevant for more practical systems and electrode geometries. The observed mass-transport effects in our system are all related to the mass-transport-sensitive HER, i.e., to pH gradients existing in the electrode boundary layer. Our work also stresses the importance of the interfacial environment, specifically for acid CO_2_ electrolysis. Tuning the interfacial environment via anion, cation, and mass-transport strategies remarkably impacts the CO2RR, as it directly influences the interfacial concentration of CO_2_. Although we realize that the flat Au model surface used in this work is very different from a practical catalyst, we nevertheless believe that the insights gained from this model system can be applied to more practical geometries and can prove valuable for industrial applications. In fact, recent GDL studies in (weak) acid have already confirmed and implemented some of the findings, such as the strategy of using concentrated weakly hydrated cations such as K^+^.^22,23^

## Supporting Information Available

Characterization of the Au ring and disk electrode for pH and FE measurements, calculation of the interfacial pH on the Au disk electrode from the peak potentials recorded during measurements, ring pH data, calculation of FE of CO2RR and HER during measurements (PDF)

## Abbreviations

CO2RR: CO_2_ reduction reaction
HER: hydrogen evolution reaction
RRDE: rotating ring-disk electrode
FE: Faradaic efficiency
