# Supplementary material for: Tuning the Interfacial Reaction Environment for CO2 Electroreduction to CO in Mildly Acidic Media
Source: J Am Chem Soc. 2024 Feb 13;146(8):5242–51. doi: 10.1021/jacs.3c11706 (PMC10910518; doi:10.1021/jacs.3c11706)
Supplement: Supplementary file 1 — ja3c11706_si_001.pdf [file ja3c11706_si_001.pdf]

Supporting Information to

Tuning the Interfacial Reaction Environment for CO_2 Electroreduction to CO in Mildly Acidic Media

Xuan Liu, Marc T. M. Koper*

Leiden Institute of Chemistry, Leiden University

2300 RA Leiden, The Netherlands

m.koper@chem.leidenuniv.nl

Characterization of the Au disk and ring electrode

The Au disk and ring electrode were characterized by cyclic voltammetry in 0.1 M H_2SO_4 respectively, to inspect possible variation of the electrode surface due to the interfacial pH measurements and the dopamine coating. As shown in Figure S1a-b, the CVs before and after dopamine coating on Au ring and disk electrode agree well with each other, signifying that dopamine residue has been totally removed and this coating process hardly influences the surface of Au ring and disk electrode. Figure s1c demonstrates that no discernable changes was traced on the surface of the Au disk during interfacial pH and FE measurements. As the peaks observed in the CVs signify the interplay between Au surface atoms, OH_{ads} and O_{ads} as well as the formation of Au oxide, they are surface sensitive. Each facet exhibits unique peak signatures. Since there is no observable deviation in the CVs, we conclude that the surface structure remains unchanged.

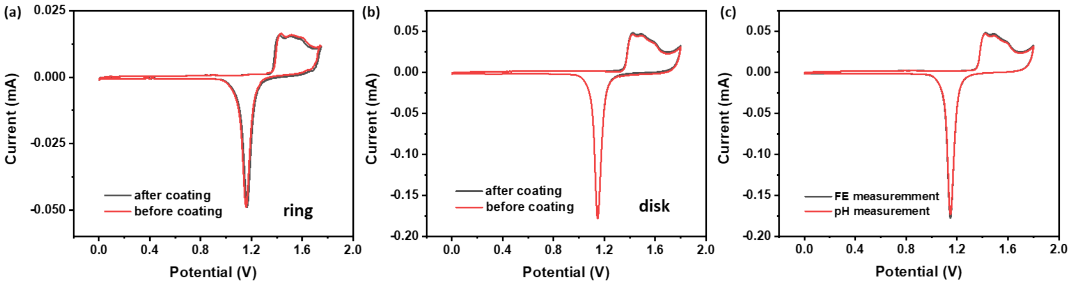

Figure S1. Cyclic voltammograms of **a)** the Au ring and **b)** the Au disk recorded in Ar-saturated 0.1 M H_2SO_4 at 100 mV s^{-1} before and after the dopamine coating. **c)** Cyclic voltammograms the Au ring and the Au disk recorded in Ar-saturated 0.1 M H_2SO_4 at 100 mV s^{-1} before the interfacial pH measurement and the FE measurement.

Calculation of the interfacial pH on the disk

Peak potentials of the 4-NSTP/4-HATP redox couple during CO2RR on the Au disk was recorded. According to the calibration curve of the redox couple (Figure S2a), the interfacial pH on the ring electrode can be obtained. To calculate the interfacial pH on the disk electrode, the relationship between pH on the ring and pH on the disk needs to be figured out. According to the convective-diffusion equation of RRDE, the ratio of the average concentration of products on the ring and the disk is defined to be the detection efficiency N_D as displayed in Eq. s2, [1] where μ is the normalized concentration of products (Eq. s1), C_∞ is the bulk concentration (The subscript "d" and "r" symbolize the ring and disk electrode, respectively). N_D is determined by the geometry of the electrode (Eqs. s3-4), in which r_1, r_2 and r_3 are the radii of the disk, the inner ring and the outer ring, respectively. [2] In this work, the N_D of the RRDE tip employed here (r_1 = 5.0 mm, r_2 = 6.5 mm, r_3 = 7.5 mm) is 0.23.

Besides, the homogenous reactions involving protons or hydroxyl ions also impact the pH gradient between ring and disk electrode. In CO_2-saturated perchlorate, when the interfacial environment turns alkaline, OH^- generated on the disk is partially consumed by either CO_2 or HCO_3^- on its way to the ring. Also, in CO_2-saturated electrolyte with protic anions, such as HSO_4^{2-} $H_2PO_4^-$ in this work, the OH^- generated by CO2R is consumed collectively by the protic anions and CO_2 and HCO_3^-.To avoid an underestimation of the interfacial pH on the disk, influences from the homogeneous buffering reactions (Eqs 2 and 4) need to be compensated.[3] For CO_2-saturated perchlorate, the normalized concentration μ here was redefined as Eq. s5 and the equation for N_D was also modified (Eq. s6). As CO_2 is continuously purged into the electrolyte during the measurements, the total carbon concentration (TC, see Eq. s7), which is the sum of the concentration of the intrinsic bicarbonate electrolyte and the saturated CO_2 concentration from extrinsic bubbling, is supposed to be constant throughout the measurements. Hence the TC for 0.1 M perchlorate under continuous CO_2 bubbling is 0.135. Concentrations of the different carbonaceous species were estimated as a function of pH (Eqs. s8-s10) by combining dissociation equilibrium constants of CO_2 (pKc_1 = 6.35, pKc_2 = 10.33). [4] As a result, the interfacial pH of the disk electrode was derived from Eqs. S6-s10. The theoretical correlation between the pH on the ring and disk in 0.1 M Sodium perchlorate is plotted in Figure S2b.

$$\mu = (C - C_\infty)/C_\infty \qquad \text{(s1)}$$

$$N_D = \frac{\mu_r}{\mu_{D,0}} = (C_r - C_\infty)/(C_d - C_\infty) \qquad \text{(s2)}$$

$$F(\theta) = \frac{3^{\frac{1}{2}}}{4\pi}\ln\left(\frac{\left(1+\theta^{\frac{1}{3}}\right)^3}{1+\theta}\right) + \frac{3}{2\pi}\tan^{-1}\left(\frac{2\theta^{\frac{1}{3}}-1}{3^{\frac{1}{2}}}\right) + \frac{1}{4} \qquad \text{(s3)}$$

$$N_D = 1 - \frac{1}{6}F\left[\left(\frac{r_2}{r_1}\right)^3 - 1\right] - \frac{2}{3}F\left[\left(\frac{r_2+r_3}{2r_1}\right)^3 - 1\right] - \frac{1}{6}F\left[\left(\frac{r_3}{r_1}\right)^3 - 1\right] \qquad \text{(s4)}$$

$$\mu_{OH^-} = (C_{OH^-} + C_{HCO_3^-} + 2C_{CO_3^{2-}} - C_{\infty,OH^-} - C_{\infty,HCO_3^-} - 2C_{\infty,CO_3^{2-}})/(C_{\infty,OH^-} + C_{\infty,HCO_3^-} + 2C_{\infty,CO_3^{2-}}) \qquad \text{(s5)}$$

$$N_D = \frac{\mu_{r,OH^-}}{\mu_{d,OH^-}} = \frac{C_{r,OH^-} + C_{r,HCO_3^-} + 2C_{r,CO_3^{2-}} - C_{\infty,OH^-} - C_{\infty,HCO_3^-} - 2C_{\infty,CO_3^{2-}}}{C_{d,OH^-} + C_{d,HCO_3^-} + 2C_{d,CO_3^{2-}} - C_{\infty,OH^-} - C_{\infty,HCO_3^-} - 2C_{\infty,CO_3^{2-}}} \qquad \text{(s6)}$$

$$TC = [CO_2] + [HCO_3^-] + [CO_3^{2-}]$$

(s7)

$$[CO_2] = \frac{TC[H^+]^2}{[H^+]^2 + K_{c1}[H^+] + K_{c1}K_{c2}} \qquad \text{(s8)}$$

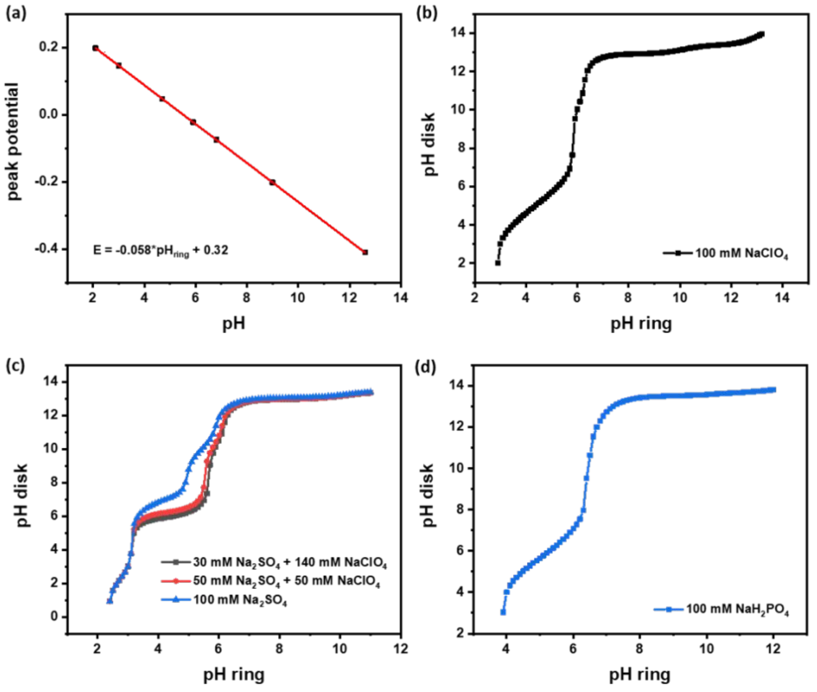

Figure S2. **a)** Calibration curve of the 4-NSTP/4-HATP redox couple measured in 0.1 M Na_2SO_4, pH is adjusted by adding H_2SO_4 or NaOH. The correspondence between pH_{ring} and pH_{disk} in CO_2-saturated **b)** 0.1 M $NaClO_4$ (pH=3), **c)** Na_2SO_4 with 200 mM Na^+ and different SO_4^{2-} concentrations (pH=3), and **d)** 0.1 M NaH_2PO_4 (pH=4).

$$[HCO_3^-] = \frac{TCK_{c1}[H^+]}{[H^+]^2 + K_{c1}[H^+] + K_{c1}K_{c2}}$$

(s9)

$$[CO_3^{2-}] = \frac{TCK_{c1}K_{c2}}{[H^+]^2 + K_{c1}[H^+] + K_{c1}K_{c2}}$$

(s10)

For CO_2-saturated sulfate, buffering from sulfuric species and carbonate species is compensated, the normalized concentration μ here was redefined as Eq. s11 and the equation for N_D was also modified (Eq. s12). TS is defined as the total concentration of sulfuric species (Eq. s13), and the concentrations of each sulfuric species were estimated as a function of pH (Eqs. s14-s15) by combining dissociation equilibrium constants of HSO_4^- (pK_{S1} = 1.99). As a result, the interfacial pH of the disk electrode was derived from Eqs. s12-s15. The theoretical correlation between the pH on the ring and disk in sodium sulfate with different SO_4^- concentration is plotted in Figure S2c.

$$\mu_{OH^-} = \frac{\left(C_{OH^-} + C_{HCO_3^-} + 2C_{CO_3^{2-}} + C_{SO_4^{2-}} - C_{\infty,OH^-} - C_{\infty,HCO_3^-} - 2C_{\infty,CO_3^{2-}} - C_{\infty,SO_4^{2-}}\right)}{\left(C_{\infty,OH^-} + C_{\infty,HCO_3^-} + 2C_{\infty,CO_3^{2-}} + C_{\infty,SO_4^{2-}}\right)}$$

(s11)

$$N_D = \frac{\mu_{r,OH^-}}{\mu_{d,OH^-}} = \frac{C_{r,OH^-} + C_{r,HCO_3^-} + 2C_{r,CO_3^{2-}} + C_{r,HSO_4^-} - C_{\infty,OH^-} - C_{\infty,HCO_3^-} - 2C_{\infty,CO_3^{2-}} - C_{\infty,SO_4^{2-}}}{C_{d,OH^-} + C_{d,HCO_3^-} + 2C_{d,CO_3^{2-}} + C_{d,SO_4^{2-}} - C_{\infty,OH^-} - C_{\infty,HCO_3^-} - 2C_{\infty,CO_3^{2-}} - C_{\infty,SO_4^{2-}}}$$

(s12)

$$TS = [HSO_4^-] + [SO_4^{2-}]$$

(s13)

$$[HSO_4^-] = \frac{TS[H^+]}{[H^+] + K_{S1}}$$

(s14)

$$[SO_4^{2-}] = \frac{TS[K_{S1}]}{[H^+] + K_{S1}}$$

(s15)

Similarly, for CO_2-saturated phosphate, buffering from phosphate species, carbonate species is compensated, the normalized concentration μ here was redefined as Eq. s16 and the equation for N_D was also modified (Eq. s17). TP is defined as the total concentration of sulfuric species (Eq. s18), and the concentrations of each sulfuric species were estimated as a function of pH (Eqs. s19-s21) by combining dissociation equilibrium constants of $H_2PO_4^-$ ($pK_{P1} = 2.14$ $pK_{P2} = 7.20$, $pK_{P3} = 12.37$). Specially, As the measurements in phosphate is performed at bulk pH 4, H_3PO_4 is ignored because of its extremely small concentration to simplify the calculation. The interfacial pH of the disk electrode was derived from Eqs. S18-s21. The theoretical correlation between the pH on the ring and disk in 0.1 M phosphate is plotted in Figure S2d.

$$\mu_{OH^-} = \frac{\left(C_{OH^-} + C_{HCO_3^-} + 2C_{CO_3^{2-}} + C_{HPO_4^{2-}} + 2C_{PO_4^{3-}} - C_{\infty,OH^-} - C_{\infty,HCO_3^-} - 2C_{\infty,CO_3^{2-}} - C_{\infty,HPO_4^{2-}} - 2C_{\infty,PO_4^{3-}}\right)}{\left(C_{\infty,OH^-} + C_{\infty,HCO_3^-} + 2C_{\infty,CO_3^{2-}} + C_{\infty,HPO_4^{2-}} + 2C_{\infty,PO_4^{3-}}\right)}$$

(s16)

$$N_D = \frac{\mu_{r,OH^-}}{\mu_{d,OH^-}} = \frac{C_{r,OH^-} + C_{r,HCO_3^-} + 2C_{r,CO_3^{2-}} + C_{r,HPO_4^{2-}} + 2C_{r,PO_4^{3-}} - C_{\infty,OH^-} - C_{\infty,HCO_3^-} - 2C_{\infty,CO_3^{2-}} - C_{\infty,HPO_4^{2-}} - 2C_{\infty,PO_4^{3-}}}{C_{d,OH^-} + C_{d,HCO_3^-} + 2C_{d,CO_3^{2-}} + C_{d,HPO_4^{2-}} + 2C_{d,PO_4^{3-}} - C_{\infty,OH^-} - C_{\infty,HCO_3^-} - 2C_{\infty,CO_3^{2-}} - C_{\infty,HPO_4^{2-}} - 2C_{\infty,PO_4^{3-}}}$$

(s17)

$$TP = [H_2PO_4^-] + [HPO_4^{2-}] + [PO_4^{3-}]$$

(s18)

$$[H_2PO_4^-] = \frac{TP[H^+]^2}{[H^+]^2 + K_{s1}[H^+] + K_{s1}K_{s2}}$$

(s19)

$$[HPO_4^{2-}] = \frac{TPK_{s1}[H^+]}{[H^+]^2 + K_{s1}[H^+] + K_{s1}K_{s2}}$$

(s20)

$$[PO_4^{3-}] = \frac{TPK_{s1}K_{s2}}{[H^+]^2 + K_{s1}[H^+] + K_{s1}K_{s2}}$$

(s21)

Ring pH data

Here we show the ring pH data corresponding to Figures 1a, 2a, 3c, 4a, 5b and 6b. The ring pH is markedly dependent on the electrolyte conditions, in agreement with the disk pH data. Noteworthy, as suggested in Figure S3a-b, the ring pH in the mass transport limited region in Na_2SO_4 is higher than that in $NaClO_4$ (and as a result, the corresponding disk pH is also higher in Na_2SO_4), illustrating the great influence of the current density on corresponding reaction environment during CO2RR. The limiting current density in Na_2SO_4 is higher due to the extra proton flux donated by HSO_4^-, leading to a less acidic reaction environment. Once the ring pH reaches around 6, as shown by the calibration curves in Figure S2, the disk pH rises quickly up to 13, due to the lack of a proper buffer anion and the weak buffer strength of the carbonaceous buffer in the electrolytes.

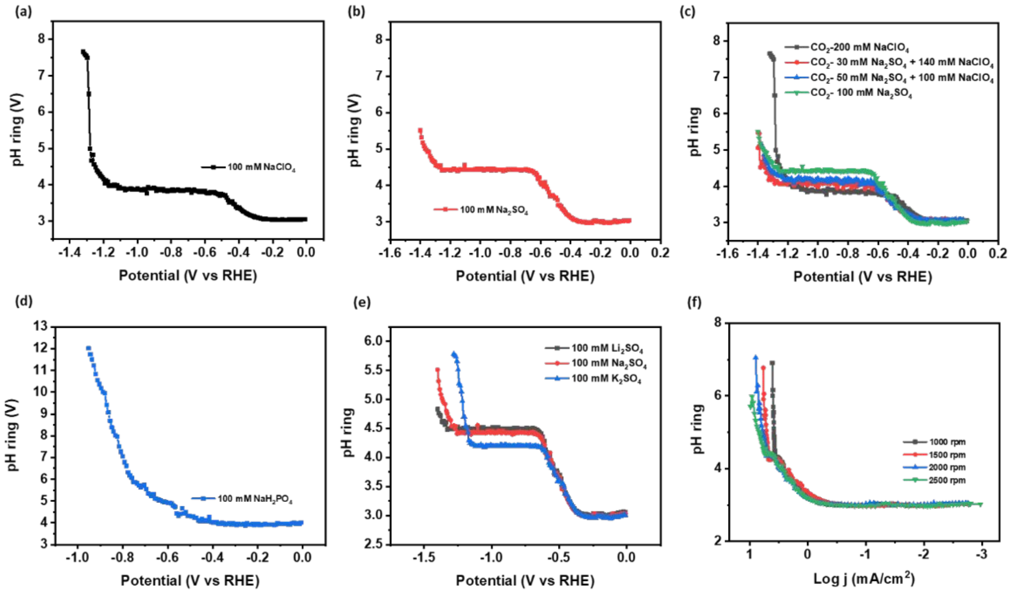

Figure S3. The ring pH data recorded during measurements in **a)** 0.1 M CO_2-saturated $NaClO_4$ with a bulk pH of 3, corresponding to the disk pH data in Figure 1a, **b)** 0.1 M CO_2-saturated Na_2SO_4 with a bulk pH of 3, corresponding to the disk pH data in Figure 2a, **c)** electrolytes with 200 mM Na^+ and different SO_4^{2-} concentrations with a bulk pH of 3, corresponding to the disk pH data in Figure 3c, **d)** 0.1 M CO_2-saturated NaH_2PO_4 with a bulk pH of 4, corresponding to the disk pH data in Figure 4a, **e)** 0.1 M CO_2-saturated sulfate with different cation identity with a bulk pH of 3 corresponding to the disk pH data in Figure 5b, **f)** 0.1 M CO_2-saturated Na_2SO_4 with a bulk pH of 3 with different rotation rates, corresponding to the disk pH data in Figure 6b. The scan rate is 2 mV s^{-1}, and the rotation rate is 2500 rpm unless specially mentioned.

Calculation of Faradaic Efficiency of CO

As a CO-producing metal, Au is also a good catalyst for CO oxidation. [5] Hence the Au ring electrode can easily deconvolute CO from other products on the disk electrode, since H_2 and CO are the only two products generated on the Au planar electrode. The partial current density for CO2RR on the Au disk can be calculated from the experimental ring current (i_{ring}) and the detect efficiency N of the Au ring (Eq. s22).

$$j_{co} = \frac{i_{ring}}{N * ECSA_{disk}} \quad \text{(s22)}$$

The Faradaic efficiency can be calculated by Eq. s23, where the i_{disk} is the current obtained on the disk during experiments.

$$FE_{co} = \frac{i_{ring}}{N * |i_{disk}|} * 100\% \quad \text{(s23)}$$

The partial current density and Faradaic efficiency of HER can be determined from Eq. s23-24

$$j_{H_2} = j_{tot} - j_{co} \quad \text{(s24)}$$

$$FE_{H_2} = 100\% - FE_{co} \quad \text{(s25)}$$

As mentioned in the experimental section, after finishing FE and interfacial pH measurements, the collection efficiency N was measured in 5 mM $K_3Fe(CN)_6$ dissolved in 0.1 M $NaHCO_3$, during which the disk was cycled from 0.27 to 1.27 V vs RHE, while the ring potential was set to 0.96 V vs RHE. The collection efficiency was determined for each rotation rate and was calculated according to Eq. s26.

$$N = \left|\frac{i_{ring}}{i_{disk}}\right| \quad \text{(s26)}$$

FE measurements during chronoamperometry

To confirm the stability of the Au ring CO sensor, FE measurements during chronoamperometry were conducted in 0.1 M CO_2-saturated Na_2SO_4, where CO2RR on the disk electrode is turned ''on'' and ''off'' at different potentials from − 0.3 V to − 1.4 V vs RHE every 200s. The rotation rate of RRDE is 2500 rpm. As depicted in Figure S4, the results agree well with the ones during cyclic voltammetry in Figure 2. FE of CO2RR increases up to ~60% along the mass transport-limited region (from − 0.6V ~ −1.2 V), and starts to decay once the water reduction sets in. During steady-state electrolysis, the ring current stays stable.

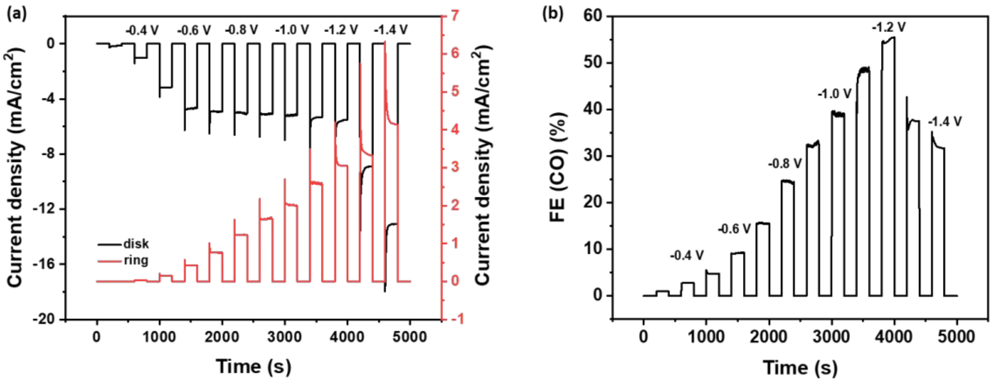

Figure S4. **a)** Current density on the disk (black curve) and ring (black curve) and **b)** FE of CO during chronoamperometry in 0.1 M CO_2-saturated Na_2SO_4 (pH=3).

Reference

(1)Albery, W., Ring-disc electrodes. Part 1.—A new approach to the theory. *Transactions of the Faraday Society* **1966,** *62*, 1915-1919.

(2)Albery, W. J.; Calvo, E. J., Ring–disc electrodes. Part 21.—pH measurement with the ring. *Journal of the Chemical Society, Faraday Transactions 1: Physical Chemistry in Condensed Phases* **1983,** *79* (11), 2583-2596.

(3)Zeebe, R. E.; Wolf-Gladrow, D., *CO2 in seawater: equilibrium, kinetics, isotopes*. Gulf Professional Publishing: 2001.

(4)Zhong, H.; Fujii, K.; Nakano, Y.; Jin, F., Effect of CO2 Bubbling into Aqueous Solutions Used for Electrochemical Reduction of CO2 for Energy Conversion and Storage. *The Journal of Physical Chemistry C* **2014,** *119* (1), 55-61.

(5)Monteiro, M. C. O.; Jacobse, L.; Koper, M. T. M., Understanding the Voltammetry of Bulk CO Electrooxidation in Neutral Media through Combined SECM Measurements. *J Phys Chem Lett* **2020,** *11* (22), 9708-9713.

(6)Blizanac, B. B.; Arenz, M.; Ross, P. N.; Marković, N. M., Surface Electrochemistry of CO on Reconstructed Gold Single Crystal Surfaces Studied by Infrared Reflection Absorption Spectroscopy and Rotating Disk Electrode. *J. Am. Chem. Soc.* **2004,** *126* (32), 10130-10141.
